# Case report: complete remission with crizotinib in *ROS1* fusion-positive sinonasal mucosal melanoma

**DOI:** 10.3389/fonc.2022.942258

**Published:** 2022-10-20

**Authors:** Jun Cao, Yaner Yu, Yangkun Zhou, Qing Ji, Wenkang Qian, Dongdong Jia, Gu Jin, Yajun Qi, Xin Li, Ningning Li, Tao Li, Meiyu Fang, Hongchuan Jin

**Affiliations:** ^1^ Sir Run Run Shaw Hospital, Medical School of Zhejiang University, Hangzhou, China; ^2^ Key Laboratory of Head and Neck Cancer Translational Research of Zhejiang Province, Department of Rare and Head and Neck Oncology, Institute of Cancer Research and Basic Medical Sciences of Chinese Academy of Sciences, Cancer Hospital of University of Chinese Academy of Sciences, Zhejiang Cancer Hospital, Hangzhou, China; ^3^ Department of Radiation Oncology, the Second Affiliated Hospital, Zhejiang University School of Medicine, Hangzhou, China; ^4^ School of Life Sciences and Technology, Southeast University, Nanjing, China; ^5^ Department of Bone and Soft Tissue Surgery, Institute of Cancer Research and Basic Medical Sciences of Chinese Academy of Sciences, Cancer Hospital of University of Chinese Academy of Sciences, Zhejiang Cancer Hospital, Hangzhou, China; ^6^ Department of Pharmacy, Institute of Cancer Research and Basic Medical Sciences of Chinese Academy of Sciences, Cancer Hospital of University of Chinese Academy of Sciences, Zhejiang Cancer Hospital, Hangzhou, China; ^7^ Department of Nuclear Medicine, Institute of Cancer Research and Basic Medical Sciences of Chinese Academy of Sciences, Cancer Hospital of University of Chinese Academy of Sciences, Zhejiang Cancer Hospital, Hangzhou, China; ^8^ Department of Pathology, Institute of Cancer Research and Basic Medical Sciences of Chinese Academy of Sciences, Cancer Hospital of University of Chinese Academy of Sciences, Zhejiang Cancer Hospital, Hangzhou, China

**Keywords:** crizotinib, ROS1, sinonasal, mucosal melanoma, complete remission

## Abstract

**Introduction:**

Sinonasal mucosal melanoma (SNMM) originates from melanocytes. Currently, the main treatment methods, including surgery, radiotherapy and chemotherapy, have little effect on the recurrence and metastasis of SNMM. However, targeted therapy may be a breakthrough in treating SNMM.

**Methods:**

A SNMM patient with ROS1 fusion received 250mg Crizotinib capsule (2 times a day, 1 tablet each time) therapy.

**Results:**

The patient achieved partial remission after 4 months of treatment and complete remission after 8 months of treatment.

**Conclusion:**

Our findings suggest that crizotinib can be an option to improve overall survival and quality of life of patients with metastatic ROS1-fusion SNMM. We believe that our report will provide insights for the application of crizotini in the treatment of melanoma.

## Introduction

Sinonasal mucosal melanoma (SNMM) is a highly invasive and racially specific malignancy arising from the melanocytes. Non-cutaneous melanocytes are found in multiple sites of human body, including the ocular tract and mucosal surfaces lining respiratory, gastrointestinal, and urogenital tracts (1). Therefore, mucosal melanoma can theoretically occur on any mucosal surface. However, the probability of carcinogenesis of mucosal melanocytes varies greatly by site, with the majority of mucosal melanomas occurring in the head and neck cavity (including oral cavity, nasal cavity, and paranasal sinuses) (31% to 55%), the rectum and anus (17% to 24%) and the female genital tract (18% to 40%) (2, 3). Furthermore, mucosal melanomas often have a cryptic origin and are often asymptomatic in their early stages. The presence of thick blood vessels at the site of the disease promoted the early metastasis and diffusion of tumor cells in lymphatic and vascular networks (4). These factors make mucosal melanoma more difficult to diagnose and treat than cutaneous melanoma. Thus, patients with mucosal melanoma (with a 5-year overall survival rate of only about 25%) have a poor prognosis compared to patients with cutaneous melanoma (with a 5-year overall survival rate of 50% to 80%) (5).

No standardized treatment regiments for SNMM has been proposed yet, and recommendations mostly emphasize treatment on a patient-to-patient basis (6). Despite the recent improvements in surgery (complete resection with clear margins is the established mainstay treatment) and chemoradiotherapy, which are frequently used in SNMM treatment, the mortality rate remains high due to local recurrence and metastasis (7, 8). Immune therapy is considered the most promising potential treatment, which has shown a significant therapeutic effect in cutaneous melanoma; however, the efficacy is lower in mucosal melanoma as the mutation patterns and susceptible population are much more heterogeneous. Meanwhile, unlike cutaneous melanoma, which are typically driven by *BRAF* and *NRAS* mutations, *SF3B1* and *KIT* have a high mutation rate in mucosal melanoma (9). Therefore, the use of targeted drugs such as imatinib (C-KIT receptor tyrosine kinase inhibitor) for treatment may become a feasible plan.

The proto-oncogene *ROS1* (ROS proto-oncogene 1, receptor tyrosine kinase) encodes a receptor tyrosine kinase whose physiological role in humans is unclear. Studies have shown that *ROS1* fusion is a significant factor driving the development of multiple cancers. *ROS1* fusion exhibit ligand-independent, constitutive catalytic activity, and these fusion activate typical cell survival and growth signaling pathways(including RAS-MEK-ERK, JAK-STAT3, PI3K-AKT-mTOR, and SHP2 cascades) (10). However, to date, no case report has mentioned *ROS1* fusion in SNMM. Herein, we report a case of SNMM with *ROS1* fusion that achieved complete remission after 8 months of crizotinib therapy. This study was approved by the Ethics Committee of Zhejiang Cancer Hospital in Hangzhou, China, and the patient signed an informed consent form.

## Case report

**Figure 1:**
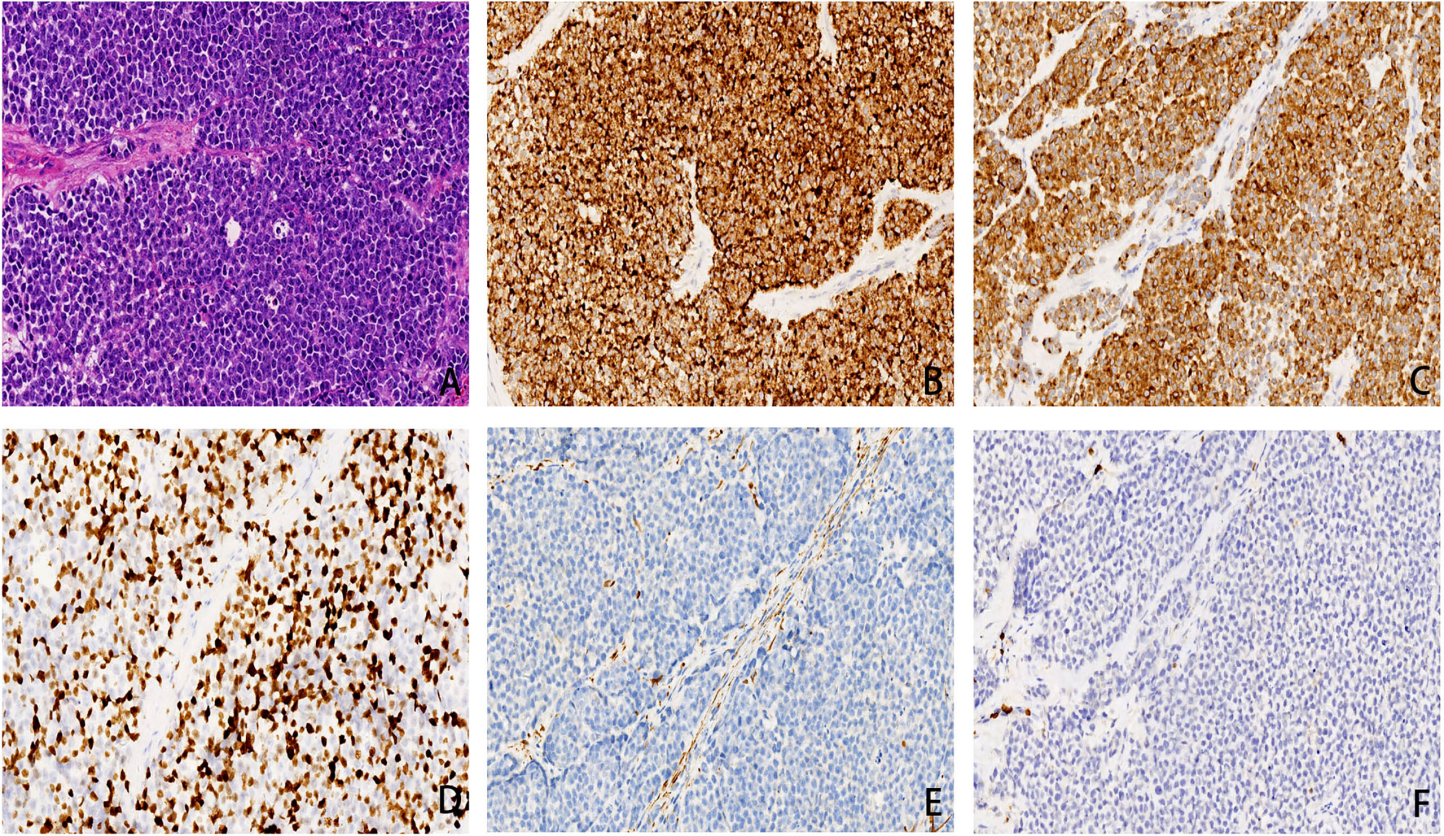
**(A)** small round cells was diffusely distributed, cellular atypia and nucleus to cytoplasm ratio were relatively high, conspicuous nucleoli and pathological nuclear division can be detected(×200 hematoxylin/eosin).**(B)** HMB45 was diffusely positive in cytoplasm, with a specificity of 100% and a sensitivity of 93%(×200 hematoxylin/eosin).**(C)** Melan-A is a marker of melanin differentiation with high sensitivity and specificity in malignant melanoma. In this case, Melan-A was diffuse positive in cytoplasm(×200 hematoxylin/eosin). **(D)** Ki67 can distinguish benign and malignant melanomas and can mark the maturation of melanin lesions. The positive cells in malignant melanomas were generally > 25%, in this case it is about 40%(×200 hematoxylin/eosin). **(E)** CK is a broad-spectrum epithelial marker. In this case, CK is negative and is distinct from epithelial carcinoma (×200 hematoxylin/eosin).**(F)** LCA is a lymphocyte marker and is negative in this case, distinguishing it from lymphoma (×200 hematoxylin/eosin).

In April 2020, a 65-year-old woman presented to the hospital with 10 months of right nasal obstruction and 1 month of recurrent bleeding. Enhanced magnetic resonance imaging (MRI) showed an irregular mass in the right nostril protruding into the nasopharyngeal cavity, suggesting the possibility of a nasal malignancy. Endoscopic nasal biopsy conformed the diagnosis of right SNMM. Right endoscopic endonasal tumor resection, septoplasty, and bottom-right turbinoplasty were performed, and the lesion was pathologically diagnosed as SNMM with necrosis and a Ki-67 positivity rate of 30%. The postoperative pathological report showed that the abnormal cell nests were found in the mucosa of the right nasal cavity. The immunohistochemistry results (positive for the expression of Ki-67, HMB45, Melan-A, S-100, and SOX10, but negative for CK, P63, EBER, EGFR and Melan-A) were consistent with malignant melanoma, and the incision margin was negative (**Figure 1**). Based on the above pathological results and clinical description, this patients tumor (T) lymph node (N) metastasis (M) staging is T3N0M0 (stage III). And no further treatment was administered after surgery. In September 2020, the woman returned to the hospital because a new mass in the right nasal cavity. Subsequently, she underwent resection of the right nasal cavity mass, and postoperative pathology indicated right SNMM with a Ki-67 positivity rate of 30%. Since ultrasound at a local hospital after surgery suggested liver metastasis, the patient presented again to our hospital.

**Figure 2:**
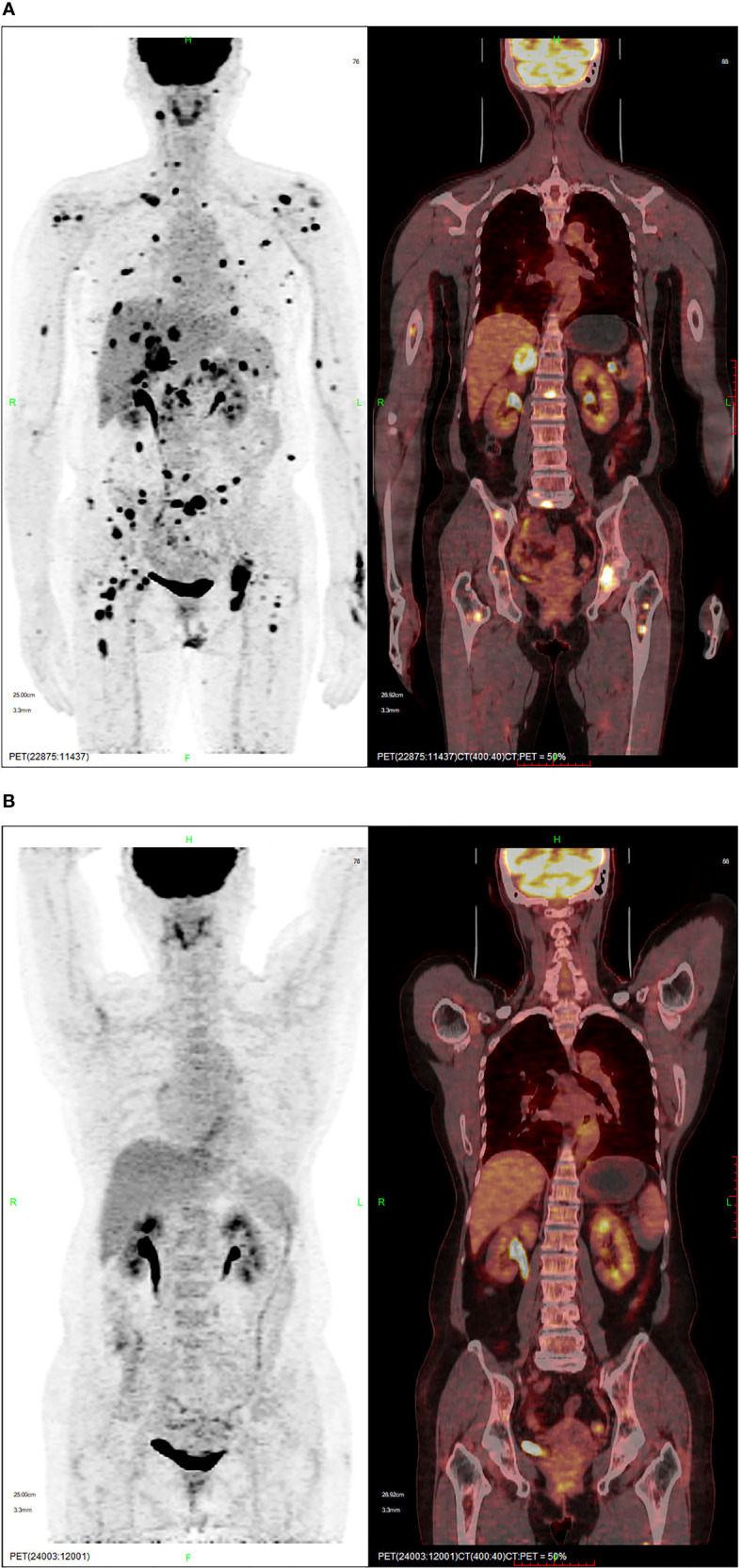
PET-CT showing **(A)** the lesion and high FDG uptake in November 2020, and **(B)** complete remission following crizotinib in August 2021.

Positron emission tomography-computed tomography (PET-CT) performed in November 2020 demonstrated high fluorodeoxyglucose (FDG) uptake in the multifocal pulmonary fields, lymph nodes, liver, intrahepatic biliary tract, pancreas, paranephros, subcutaneous tissue, muscles, and bone, indicating tumor metastases (**Figure 2A**). Chemotherapy would be the rational standard therapy; however, this patient had a poor constitution and systemic metastasis. Additionally, a clinical trial from Beijing Cancer Hospital suggested that axitinib combined with toripalimab was tolerable and showed promising antitumor activity in patients with treatment-naïve metastatic mucosal melanoma (11). Therefore, patient was initially treated with immunotherapy combined with targeted therapy (240 mg triprizumab once every 3 weeks, and 5 mg axitinib twice daily); however, the result was not satisfactory and the progression-free survival (PFS) was 1.4 months. During the treatment, she experienced the side effect of diarrhea, hand-foot syndrome, and both of which belonging to the Common Terminology Criteria for Adverse Events 5.0 (CTCAE 5.0) grade 1. Computed tomography (CT) demonstrated tumor progression in both lungs, liver, paranephros, and abdominal subcutaneous tissue in December 2020.

**Figure 3:**
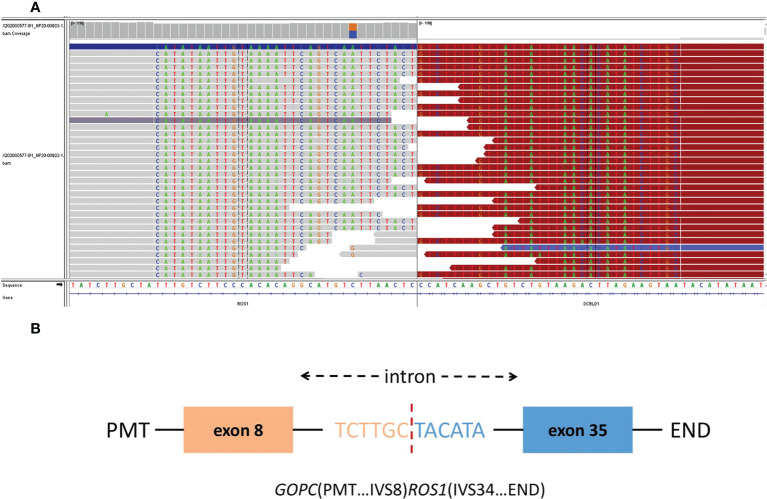
The Integrative Genomics Viewer Screenshot **(A)** of GOPC-ROS1 gene fusion are displayed by whole exome sequencing. The schematic diagram **(B)** represents the GOPC-ROS1 fusion protein domain structure.

On whole exome sequencing (WES), *GOPC-ROS1* fusion and *ERBb2* overexpression were found (**Figure 3**). Consequently, the patient was given 250 mg crizotinib capsule (2 times a day, 1 tablet each time) from December 2020. Before this treatment, she had a poor physical condition and needed prolonged bedrest; after 1 month of crizotinib treatment, the patient was able to do some laundry and her spirits lifted. She experienced the side effect of diarrhea, hypohepatia, and hyperthyroidism, all of which belonging to the CTCAE grade 1. Abdominal and chest CT scans performed in April 2021 showed no obvious space-occupying lesions in the nasal cavity, and the remaining lesions were reduced, indicating PR (partial response) with a >30% decrease in the sum of the lesion diameters compared to previous examinations. A PET-CT in August 2021 showed complete regression of the lesions (**Figure 2B**).

**Figure 4:**
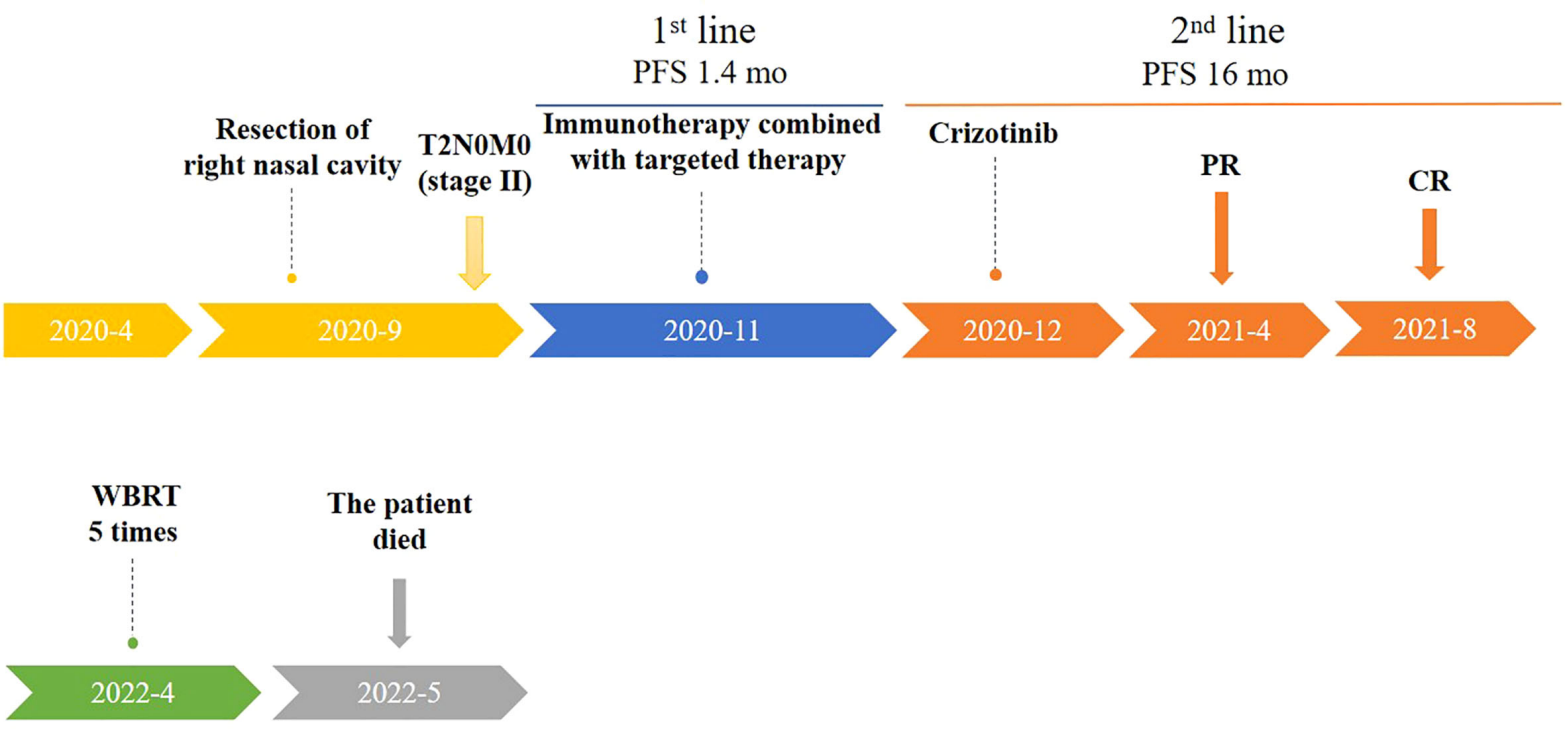
Timeline of patient treatment (from April 2020 to May 2022). Treatment process and effect evaluation of the patient. PFS,Progression-free Survival; mo, months; PR, Partial Response; CR, Complete Regression; WBRT, Whole Brain Radio Therapy.

The patient presented with walking instability in March 2022. MRI of brain on April 15, 2022 showed multiple abnormal enhancement images in both cerebral hemispheres, cerebellum and brainstem, with obvious enhancement after enhanced, the largest was about 1.4cm, and no edema signal was observed around. Therefore, metastatic tumor was considered. On April 20, 2022, the patient received whole brain radio therapy after admission the target areas included the whole brain and metastases [D95: PTV400cGy/20F (metastases), PTVm5000cGy/20F (whole brain)]. After 5 times of radiotherapy, the patient’s eating, swallowing function and muscle strength decreased. After treatment with dehydrating hormone, the patient did not improve significantly, so radiotherapy was stopped. Subsequently, the patient made the decision to stop treatments and deceased due to her cancer on 12 May 2022 (**Figure 4**).

## Discussion

*ROS1* fusion is mostly detected in non-small cell lung cancer (NSCLC), but it is also detected in other neoplasms, including myofibroblastic tumors, stomach adenocarcinoma, cholangiocarcinoma, and glioma (12, 13). *ROS1* fusion is present in 9% of all spitz neoplasms, a subtype of melanomas, where it strongly activates the MAPK/ERK and PI3K/AKT/mTOR pathways (14, 15). Compared to non-*ROS1* fusion tumors, *ROS1* fusion-positive spitz neoplasms are less likely to have high-grade nuclear atypia (P=0.006), the tumor cell size is small to intermediate (P = 0.001), and there is a tendency for a lower mitotic rate of 1.3/mm ^2^ (P =0.001), all suggesting an indolent or low grade tendency (16). Only two cases have been reported with the *ROS1* fusion in melanomas other than spitz neoplasms. Turner (17) reported a *ROS1* rearrangement in a superficial spreading melanoma which was negative for ROS1 protein expression, but targeted RNA sequencing and quantitative Real-time PCR (real-time qPCR) consistently confirm this mutation. Drilon (18) reported another case of *ROS1*-rearranged acral lentiginous melanoma with partial response to entrectinib, a tyrosine kinase inhibitor (TKI) with good brain barrier penetration ability; this patient remains on entrectinib therapy at 12 months as of the data cutoff date.

Crizotinib is an effective and economical TKI targeting *ROS1* in NSCLC. It has a high objective response rate (ORR, approximately 70–80%) and manageable side effects, including visual impairment, nausea, peripheral edema, complex hepatic cysts, QT-prolongation, and liver enzyme elevation (19, 20). It has an ORR of 86.7% versus 44.7% for platinum-pemetrexed chemotherapy as first-line therapy for lung cancer, and prolonged the median progression-free survival (PFS) to 18.4 months compared to the chemotherapy group (8.6 months) in a large-scale clinical trial on *ROS1*-fusion NSCLC (21). Despite extensive use in NSCLC, this drug’s effectiveness in melanoma has just begun to be explored. Oliver (22) developed a uveal melanoma metastatic mouse model and showed that although crizotinib has marginal effects on tumor growth inhibition, it has an important role in preventing the development of metastatic spots in uveal melanoma xenografts. However, the latest phase II multicenter trial of adjuvant crizotinib in high-risk patients revealed different results, In this trial, adjuvant therapy with crizotinib showed no significant difference in recurrence free survival (RFS) from that with control arm, and 11/34 (32%) patients experienced CTCAE grade 3 or 4 AE (23). The conclusion of this paper is quite different from that of the above report, which reveals the limitation of the mouse model. However, in view of the fact that this manuscript is a case report involving only one patient and the particularity of this patient, there are still some differences between our report and clinical trials. Das (24) found that the combination of crizotinib and apatinib can attenuate the viability of melanoma cells, decrease cell proliferation, and increase cell apoptosis, and these combined effects are speculated to block the pathways that are utilized by cutaneous malignant melanoma cells to counteract single drug-induced cell death. Moreover, in spitzoid melanomas, increased phosphorylation of the PWWP2A–ROS1 chimeric protein (which is most frequently seen in spitz neoplasms) was detected in melanocytes and it is at least partially inhibited by crizotinib (15), although its effect on mucosal melanoma remains unknown.

An ASO Author Reflections (25) from MD Anderson Cancer Center referred to the promising results of immunotherapy in patients with mucosal melanoma, especially the encouraging clinical response to TKIs in patients with advanced melanoma harboring genetic aberrations. Our case is a special and successfully treated example, since this patient was not positive for the most frequent biomarkers: *NRAS, KIT*, and *BRAF*. We have described a case with a rare mutation in a rare tumor and shown that individualized treatment regimens are crucial for providing optimized symptom control. Our report demonstrates that crizotinib is an option for ROS1-driven malignancies. In addition, for patients with malignant melanoma, BRAF or c-KIT test alone is not enough. It is suggested that WES should be carried out when conditions permit, so as to obtain specific targets for precise treatment. Furthermore, genetic testing is very important to confirm the diagnosis and prognosis as well as to guide treatment.

Since the *NTRK* basket trial demonstrates the efficacy of entrectinib as a monotherapy in metastatic *NTRK* rearranged solid tumors (26). Basket trails can be attempted for *ROS1* rearranged or *ROS1* fusion tumors, in which *ROS1* inhibitors such as crizotinib are used whenever *ROS1* rearranged or *ROS1* fusion is detected, regardless of tumor types, for a broader tumor agnostic approval. Furthermore, if crizotinib is approved as a first-line drug, it will replace the combination of immunotherapy and axitinib, and patients will have better prognosis.

The National Comprehensive Cancer Network (NCCN) guidelines recommend adjuvant immunotherapy for patients with T3N0 stage nasal mucosal malignant melanoma. If not receiving adjuvant immunotherapy, at least local radiotherapy is also given (27). However, since the patient was first diagnosed in an ophthalmology and otorhinolaryngology hospital, rather than a cancer hospital, and due to the doctor’s insufficient understanding of the tumor, the patient was not advised to receive subsequent adjuvant treatment, which is very regrettable. Therefore, we recommend that patients with these rare tumors need specialized treatment in cancer hospitals.

## Data availability statement

The original contributions presented in the study are included in the article/Supplementary Material. Further inquiries can be directed to the corresponding authors.

## Ethics statement

The studies involving human participants were reviewed and approved by the Ethics Committee of Zhejiang Cancer Hospital in Hangzhou, China. The patients/participants provided their written informed consent to participate in this study. Written informed consent was obtained from the individual(s) for the publication of any potentially identifiable images or data included in this article.

## Author contributions

JC: Conceptualization, Validation, Writing-original draft, Writing- review & editing. YY: Writing - original draft. QJ: Investigation, Data curation. WQ: Data curation. DJ: Investigation, Data curation, Visualization. GJ: Validation, Writing - original draft. YQ: Writing-original draft. XL: Imaging data collection. NL: Pathological data collection. TL: Resources, Supervision. MF: Conceptualization, Resources, Writing-review & editing, Supervision. HJ: Writing-review & editing, Supervision. All authors contributed to the article and approved the submitted version.

## Funding

This study was supported by National Natural Science Foundations of China (81702653 to JC) and Health high-level personnel training project (CZ403).

## Conflict of interest

The authors declare that the research was conducted in the absence of any commercial or financial relationships that could be construed as a potential conflict of interest.

## Publisher’s note

All claims expressed in this article are solely those of the authors and do not necessarily represent those of their affiliated organizations, or those of the publisher, the editors and the reviewers. Any product that may be evaluated in this article, or claim that may be made by its manufacturer, is not guaranteed or endorsed by the publisher.
